# Structural Optimization and Finite Element Analysis of Variable-Stiffness Biodegradable Vascular Stents

**DOI:** 10.3390/jfb17060296

**Published:** 2026-06-14

**Authors:** Hanbing Zhang, Shiliang Chen, Tianming Du, Yanping Zhang, Lifang Wu, Aike Qiao

**Affiliations:** 1 School of Information Science and Technology, Beijing University of Technology, Beijing 100124, China; 2 College of Chemistry and Life Science, Beijing University of Technology, Beijing 100124, China

**Keywords:** biodegradable vascular stent, finite element analysis, non-uniform degradation, stress corrosion cracking

## Abstract

Premature structural failure of biodegradable vascular stents (BVSs) induced by stress corrosion cracking (SCC) remains a critical challenge. Heterogeneous plaques compress the stent, leading to inadequate expansion and inducing stress concentration that exacerbates SCC. This study proposes variable-stiffness stents to improve radial support and mitigate non-uniform degradation. The stents were designed with shortened axial ring segments and selective strut widening at the stenotic regions for targeted stiffness enhancement. They were virtually deployed in arteries with non-calcified and calcified plaques to evaluate immediate performance, while long-term service behavior was assessed via degradation simulation under combined electrochemical corrosion and SCC effects. The results show that variable-stiffness stents exhibited comparable residual stenosis to uniform-stiffness stents with identical local structures at plaque regions. Dual-stiffness designs yielded a smoother luminal profile than uniform-stiffness counterparts, and gradient-stiffness designs achieved further improvements. Local strut widening extended full recoil time, with a more marked effect on high-stiffness segments, by 52.6% and 41.2% in non-calcified and calcified plaques, while simultaneously increasing volume loss to mitigate non-uniform degradation. In addition, widened gradient-stiffness designs further prolonged the stable support time by 9.7%. These findings show variable-stiffness stents with widened gradient-stiffness design exhibit a more favorable immediate and long-term performance.

## 1. Introduction

Percutaneous coronary intervention with stent implantation rapidly revascularizes stenotic arteries, representing a highly effective therapy for coronary artery disease [1]. Biodegradable vascular stents (BVSs) provide initial mechanical support comparable to that of permanent drug-eluting stents, but are designed to gradually degrade upon the completion of vascular remodeling [2]. This temporary scaffolding aims to mitigate potential long-term complications associated with permanent stents, such as in-stent restenosis (ISR) and late stent thrombosis (LST) [3].

Following implantation, the stent degradation kinetics dictate its long-term mechanical stability and clinical safety. However, both clinical observations and computational simulations have revealed that BVS degradation is spatially non-uniform, frequently manifesting as early localized strut fractures [4,5]. Such premature loss of mechanical integrity triggers structural collapse, severely compromising the therapeutic efficacy.

BVS degradation is driven by both general and localized corrosion [5]. General corrosion is an electrochemical process that occurs uniformly across the stent surface and dictates the baseline degradation rate. However, localized corrosion is the primary driver of non-uniform degradation, which mainly manifests as pitting corrosion and stress corrosion cracking (SCC). Pitting corrosion is associated with surface defects and can be mitigated by advanced manufacturing and optimized deployment protocols [5]. SCC is triggered by the synergistic interaction between localized tensile stress and the physiological blood environment [6]. It remains a formidable challenge.

The initiation and propagation of SCC depend heavily on the magnitude and spatial distribution of post-deployment stress. In clinical practice, stents are deployed into stenotic arteries, where the specific composition and geometry of atherosclerotic plaques dictate this stress field. Heterogeneous plaques exert severe compression and induce stress concentrations across the stent, especially when highly stiff calcified plaques are involved [4]. Once SCC initiates in these high-stress regions, it further amplifies local stress concentrations due to the compromised strut cross-section. This intensified stress then accelerates subsequent SCC progression, creating a vicious cycle that ultimately culminates in premature strut fracture [7].

To combat these stress-driven failures, considerable efforts have been devoted to material optimization. Biodegradable zinc (Zn) alloys have emerged as promising candidates for BVSs due to their appropriate degradation rates and biocompatibility [8]. While pure Zn exhibits low SCC susceptibility, its limited mechanical strength necessitates alloying for enhancement [9]. Microalloying with elements such as Mn, Li, Cu, or Ag was employed to significantly improve both strength and ductility [10,11,12,13]. Although current research did not explicitly indicate that alloying induces high SCC susceptibility, a residual vulnerability of SCC-related cracking invariably emerges [9]. Particularly when subjected to the complex biomechanical and physiological demands of the in vivo environment, SCC remains an unavoidable failure risk that must be rigorously managed.

Beyond material selection, structural optimization is essential to simultaneously mitigate local stress concentrations and enhance radial support. To alleviate localized stress, smooth cell geometries and rounded connectors have been adopted [14]. Furthermore, to facilitate a more uniform stress distribution, researchers have employed advanced architectures such as auxetic lattices leveraging negative Poisson’s ratios [15], sinusoidal or arc-shaped struts [16], and optimized connector topologies. Concurrently, to enhance radial strength and resist structural collapse, recent studies have introduced novel geometric configurations, including interlocking tenon-and-mortise structures [17], short strutting rings integrated within links [18], and reverse-aligned or unequal-height support rings [19]. Extensive finite element analysis (FEA) has confirmed the enhanced biomechanical efficacy of these structural optimizations.

However, these optimized designs still adopt a globally uniform stiffness strategy. Although effective under normal physiological conditions, this approach lacks adaptability to heterogeneous plaque characteristics. Increasing the overall radial strength to dilate the central stenosis often results in over-expansion at the adjacent healthy arterial edges. Furthermore, these initial geometric distortions directly translate into concentrated local stresses that accelerate SCC. To alleviate this initial deployment mismatch, spatially modulating stiffness along the stent axis has been proposed [20]. Yet, further investigations into applying this strategy to BVSs remain lacking.

Beyond this spatial limitation, a temporal disconnect exists in current BVSs research. Studies on structural optimization generally regarded stent deployment as an independent mechanical process, while neglecting the influencing effect of the post-deployment stress field on the long-term degradation of biodegradable stents. Conversely, studies dedicated to simulating BVS degradation mainly relied on idealized artery models or focus on single stent rings [5,21]. This oversimplification largely stems from the prohibitive computational expense of simulating the highly non-linear mechanics between the stent and the stenotic artery throughout the long-term degradation process of a full-scale structure. Because these simplified models artificially generate a uniform stress distribution, they fail to capture the complex degradation behaviors within stenotic lesions. Consequently, the interaction between the immediate post-deployment mechanics and the long-term degradation remains to be explored.

**Figure 1:**
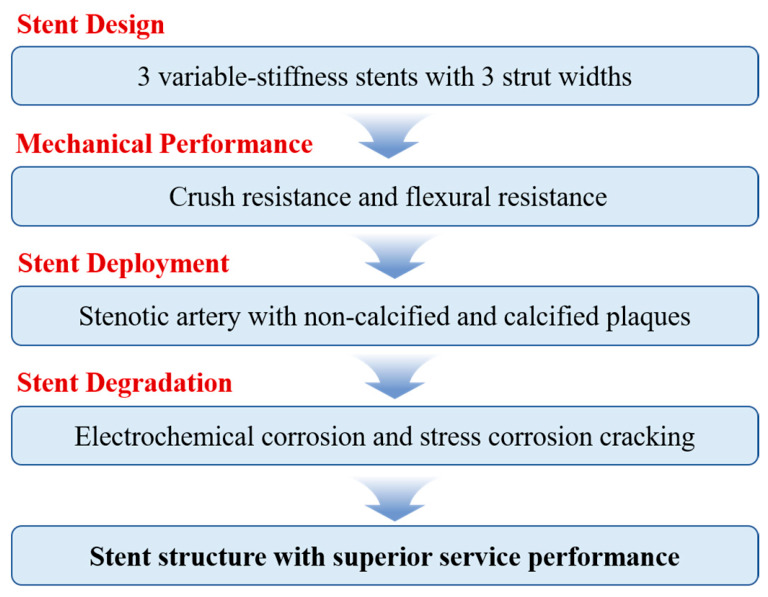
Structural optimization workflow of variable-stiffness stent.

To address these critical issues, this study proposes a variable-stiffness zinc alloy stent designed to simultaneously enhance radial support for stenotic arteries and mitigate non-uniform degradation caused by stress concentrations (Figure 1). Within the numerical simulation framework, the mechanical performances are first compared against three corresponding traditional uniform-stiffness stents, followed by the virtual deployment of these stents into stenotic arteries with varying plaque properties. Subsequently, a computationally efficient degradation model is developed to simulate the corrosion behavior of the full-scale stents. Ultimately, based on these simulations, the optimal stent structures with both immediate and long-term performance are identified.

## 2. Materials and Methods

### 2.1. Stent Design

**Figure 2:**
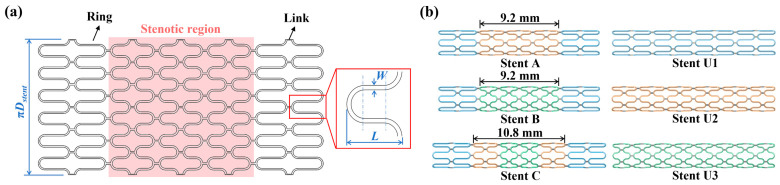
Geometry model of variable-stiffness stent: (**a**) 2D unrolled view of the stent; (**b**) Three types of variable-stiffness stents and three corresponding uniform stiffness stents (where blue, yellow and green rings represent axial lengths *L* of 1.4 mm, 1.2 mm, and 0.8 mm, respectively).

As depicted in the 2D unrolled view (Figure 2a), the local radial stiffness enhancement was achieved by shortening the axial ring length (*L*) and selectively increasing the strut width (*W*) at the central stenotic region. These rings were interconnected by straight, I-shaped links of a consistent length. Based on this concept, three variable-stiffness stents were designed: Dual-stiffness Stents A and B consisted of longer 2.0 mm rings at both ends and shorter rings at the center, measuring 1.2 mm for Stent A and 0.8 mm for Stent B (Figure 2b). In contrast, gradient-stiffness Stent C featured a length transition from 2.0 mm at the ends, through 1.2 mm, to 0.8 mm at the center, ensuring a gradual stiffness variation along the axial direction.

To further enhance central support and mitigate SCC during degradation, the struts of the central higher-stiffness rings were locally reinforced. Building upon a baseline strut width of *W*1 = 0.10 mm, two widened configurations of *W*2 = 0.12 mm and *W*3 = 0.14 mm were implemented for the rings positioned at the central stenotic region. All stent models were constructed with an initial outer diameter of 3.0 mm. The total axial length was maintained at 18.8 mm for all designs, with the exception of Stent C, which was slightly extended to 20.4 mm to accommodate the gradient transition of ring lengths. To ensure comparability in subsequent corrosion simulations, the variation in specific surface area among stents with identical central widths was controlled within 0.6% (Table 1). For comparative analysis, three traditional uniform-stiffness stents (Stents U1, U2, and U3) featuring a consistent 0.10 mm ring width across their entire length were also modeled (Figure 2b).

### 2.2. Mechanical Performance

#### 2.2.1. Crush Resistance

**Figure 3:**
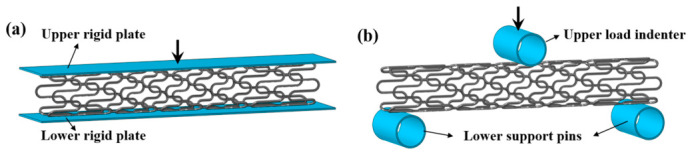
Stent mechanical properties test and stent deployment: (**a**) Crush resistance; (**b**) Flexural resistance.

To evaluate the basic mechanical performance of the designs, a parallel-plate compression simulation was performed (Figure 3a). Each stent was positioned horizontally between two analytical rigid plates. While the lower plate was fully constrained in all degrees of freedom, a prescribed downward displacement was applied to the upper plate to induce progressive compression [22]. This process continued until the outer diameter of the stent reached 50% of its initial value. The total reaction force acting on the upper plate was extracted to quantify the radial resistive force (RRF) for each stent design.

#### 2.2.2. Flexural Resistance

To assess the bending flexibility, a three-point bending simulation was conducted (Figure 3b). Each stent was positioned horizontally across two rigid cylindrical support pins separated by a span length of 16.8 mm. Consistent with the crush resistance setup, the lower support pins were fully constrained in all degrees of freedom, while a prescribed downward displacement was applied via a central rigid indenter to induce progressive bending [22]. This deformation process continued until the central indenter reached a target displacement of 3.0 mm. The total reaction force extracted from the central indenter was utilized to quantify the flexural resistive force (FRF) of the stent.

#### 2.2.3. Material Properties

The stent was assigned the material properties of a novel biodegradable zinc-manganese (Zn-Mn) alloy developed by our collaborators. The Zn alloy was modeled as a homogeneous and isotropic elastoplastic material. The elastic behavior was characterized by a Young’s modulus of 97 ± 14 GPa and a Poisson’s ratio of 0.27. The plastic deformation was described using isotropic hardening based on *J*_2_ flow theory, incorporating true stress–strain data derived from experimental uniaxial tensile tests. Specifically, the material exhibited a yield strength of 323 ± 11 MPa, and ultimate tensile strength (UTS) of 401 ± 14 MPa.

### 2.3. Stent Deployment

#### 2.3.1. Artery Model

**Figure 4:**
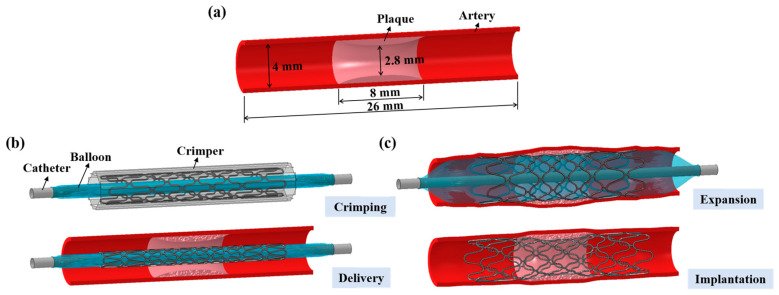
Stent deployment: (**a**) Artery geometry; (**b**) Stent crimping and delivery; (**c**) Stent expansion and implantation.

The arterial model was constructed as a cylindrical segment with an inner diameter of 4.0 mm (Figure 4a). To simulate the stenotic state after pre-dilatation, an atherosclerotic plaque with a 30% diametric stenosis was incorporated at the mid-span of the artery. The axial length of the plaque was set slightly shorter than the high-stiffness region of the stent, ensuring that the lesion was completely covered by its high-support central section. The artery was modeled as an isotropic incompressible hyperelastic material. Its highly nonlinear behavior was captured using a third-order Ogden model [23]. To investigate the impact of different lesion types on the service performance of variable-stiffness stents, both non-calcified and calcified plaque models were employed. Their respective mechanical behaviors were described using a first-order Ogden model, with material constants defined to reflect the differences in tissue stiffness (Table 2) [23,24].

#### 2.3.2. Crimping and Delivery Behavior

The stent crimping process was simulated by imposing inward radial displacements to eight rigid crimpers circumferentially distributed around the stent to gradually reduce the stent outer diameter (Figure 4b) [22]. Subsequently, the crimpers were retracted, allowing the stent to undergo slight elastic recoil and securely engage with the underlying balloon–catheter assembly. Finally, the crimped stent-balloon system was coaxially positioned within the lumen and delivered to the stenotic lesion.

#### 2.3.3. Expansion and Deflation Behavior

The stent expansion was achieved by applying an internal pressure of 10 atm to the inner surface of a non-compliant balloon, dilating the stenotic lumen until it reached 1.1 times the diameter of the adjacent healthy artery (Figure 4c) [23]. Following a brief holding step at maximum expansion, the balloon was deflated, allowing the stent to undergo free elastic recoil and achieve its final implantation state. Then the balloon was withdrawn from the model. Ultimately, the stent stress field was obtained, and the residual stenosis and axial lumen variation were extracted to evaluate the immediate efficacy of the deployment.

### 2.4. Stent Degradation

**Figure 5:**
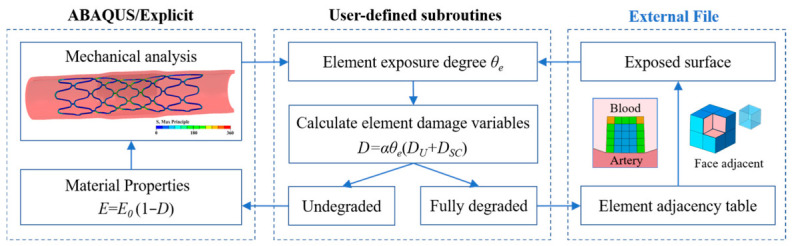
Framework of stent degradation.

From a macroscopic perspective, stent degradation manifests as a dynamic process characterized by the deterioration of material properties and volume loss due to corrosion in the physiological bloodstream environment. To computationally capture these physical changes, a phenomenological dynamic degradation model was established (Figure 5).

#### 2.4.1. Material Damage Evolution

Based on continuum damage mechanics (CDM), the influence of microscale material discontinuities on the macroscopic mechanical integrity was characterized by a dimensionless scalar damage variable, *D* [5]. As degradation progressed, *D* monotonically increased from 0 to 1, representing the material transition from an undamaged state to a fully degraded state. According to the strain equivalence principle, the material properties of the stent were linearly attenuated by *D*. Specifically, the degraded Young’s modulus was calculated as Equation (1) [21]:

*E* = *E*_0_(1 − *D*), (1)

where *E*_0_ denotes the initial Young’s modulus of the undamaged zinc alloy, and *E* is the effective modulus at the current degradation state. To prevent numerical singularities during the explicit finite element analysis, the Young’s modulus of fully degraded elements was set to a residual value of 0.001 MPa prior to their deletion from the computational mesh.

The evolution of total damage was formulated to capture the synergistic effects of uniform electrochemical corrosion and SCC. Assuming a linear superposition of these two primary mechanisms, the localized degradation rates were further accelerated by hemodynamic shear stress. Consistent with existing literature, this flow-induced acceleration effect was positively correlated with the element exposure degree [25]. Therefore, the accumulation of total damage was formulated as Equation (2) [26]:

*D* = *αθ_e_*(*D_U_* + *D_SC_*), (2)

where *α* denotes the time relaxation factor governing the degradation progression of the stent, *θ_e_* characterizes the degree of element exposure, and *D_U_* and *D_SC_* represent the specific damage variables attributed to uniform corrosion and SCC, respectively.

Within this framework, *D_U_* was calibrated against a representative in vitro immersion corrosion rate of 0.18 mm/year [5,27]. In contrast, the stress-driven damage component, *D_SC_*, was activated exclusively when the local stress exceeded a stress-corrosion threshold. Due to the scarcity of specific stress-corrosion thresholds for zinc alloys, reasonable phenomenological assumptions were adapted from more extensively studied magnesium alloys, which share fundamentally similar computational logic for electrochemical corrosion and stress-accelerated damage [28]. Accordingly, the stress-corrosion threshold was defined as 50% of the yield strength [29]. The specific partial differential equations governing the evolution of these damage variables have been detailed in our previous work [5]. Once the total accumulated damage reached the fully degraded state, the corresponding element was deleted from the model.

#### 2.4.2. Geometric Evolution

Physically, the corrosion of the alloy stent is an interfacial phenomenon, occurring exclusively at the exposed surfaces. To computationally capture this moving boundary, a dynamic surface tracking algorithm was implemented (Figure 5). After spatial discretization, a global element adjacency table was established. For the employed hexahedral elements, the specific exposure degree was quantified by the number of their exposed faces. Elements directly interacting with the blood environment immediately post-deployment were defined as the initial exposed surface [5]. Upon the deletion of a fully degraded element, the algorithm queried the adjacency table to update the exposed surface, and the exposure degrees of the face-adjacent elements were immediately recalculated. The element adjacency table and initial exposed surface were recorded in external data files.

#### 2.4.3. Dynamical Degradation

The stress field and deformed geometry obtained after stent deployment served as the initial state for the stent degradation. As the stent progressively degraded, its material properties and exposed surfaces were continuously evolved, resulting in a dynamic redistribution of stress field. The stress-corrosion threshold, which is dependent on the local material properties, shifted accordingly. Consequently, both this updated stress state and the locally attenuated material properties interactively dictated the subsequent degradation evolution [26]. Therefore, a time-dependent iteration in the FEA was performed to achieve a new mechanical equilibrium state at each increment (Figure 5).

To evaluate the long-term service performance, both the global stent volume loss and the corresponding arterial radial recoil at the central stenotic region were continuously monitored. The volume loss at the moment when the artery recoiled to its pre-deployment state was utilized as the quantitative metric for assessing degradation uniformity. The volume loss was formulated as Equation (3):

(3) $$volume\;loss=\frac{V_{stent}-\sum \left({L_{e}}^{3}\cdot D_{i}\right)}{V_{Stent}}\times 100\%,$$

where *L_e_* represents the characteristic length of the hexahedral element, *D_i_* denotes the dimensionless damage variable of the stent *i*-th element and *V_stent_* corresponds to the initial total volume of the stent.

### 2.5. Computational Simulation

#### 2.5.1. Meshing

For spatial discretization, a mesh sensitivity analysis was performed independently for each component to ensure a balance between computational accuracy and efficiency. Taking the stent as a representative example, the mesh density was progressively refined by increasing the number of element layers along the thickness direction. The optimal element sizes were determined when the variation in the maximum principal stress at the strut crowns between successive mesh refinements fell strictly below 3%.

Based on these results, the artery and stent were meshed using C3D8R hexahedral elements with characteristic lengths of 0.06 mm and 0.02 mm, respectively. The plaque was discretized with C3D4 tetrahedral elements assigned a characteristic length of 0.02 mm, while the balloon was modeled using S4 shell elements with a length of 0.04 mm. Subsequently, the overall mesh quality was thoroughly verified.

#### 2.5.2. Boundary Conditions

A general contact was defined among the testing fixtures, stent, stenotic artery, and balloon, utilizing hard contact for the normal behavior and a penalty formulation with a friction coefficient of 0.2 for the tangential behavior [30]. A tie constraint was imposed between the plaque and the artery to represent their physiological bonding. Throughout both the stent deployment and subsequent degradation phases, axial displacement constraints were applied to both ends of the artery to simulate the tethering effect of the surrounding vascular tissue [31].

#### 2.5.3. Finite Element Analysis

All numerical simulations were performed using the AbaqusExplicit solver (version 2020; Dassault Systèmes SE, Vélizy-Villacoublay France). The stent degradation was simulated by extending the solver via user-defined subroutines VEXTERNALDB and VUSDFLD. VEXTERNALDB was utilized to manage the external data files and to synchronize the start and end of each increment. In VUSDFLD, solution-dependent state variables (SDVs) were defined to continuously record the instantaneous degree of element exposure, stress states, and cumulative damage of the stent elements. The field variables were then updated according to SDV evolution to dynamically adjust material properties for degrading elements.

To improve computational efficiency, a multi-threaded parallel strategy was adopted within the subroutines. The external files were read only once during initialization and stored as global variables in a Fortran COMMON block to avoid repeated file I/O. Thread-local variables were used to record SDVs and field variables for elements processed by each thread. When an element was deleted, the program accessed the COMMON block and updated the exposed surface states of neighboring elements via the adjacency table. Mutex locks were strictly applied to ensure thread safety during concurrent access to these data.

To minimize dynamic effects and ensure a quasi-static response during the stent implantation and degradation processes, a Rayleigh damping coefficient (α = 8000) was applied to the artery. By controlling energy dissipation, this parameter effectively prevented unrealistic fluctuations in the arterial diameter, maintaining the ratio of kinetic energy to internal energy strictly below 5% throughout the entire analysis.

## 3. Results

### 3.1. Evaluation of Mechanical Performance

**Figure 6:**
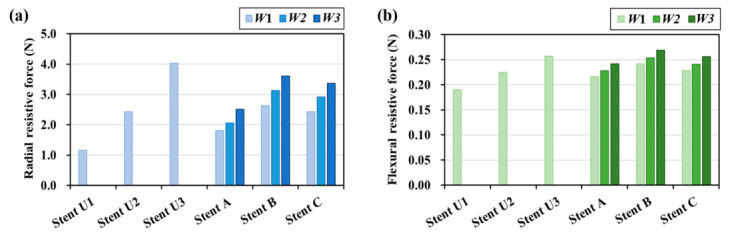
Comparison of stent mechanical performances: (**a**) Crush resistance; (**b**) Flexural resistance.

For the uniform-stiffness designs, decreasing the axial ring length from Stent U1 to U3 increased the global RRF from 1.16 N to 4.03 N (Figure 6a). However, this came at the expense of flexibility, evidenced by an increase in the FRF from 0.19 N to 0.26 N (Figure 6b). By combining rings of different stiffness, the dual-stiffness designs exhibited intermediate mechanical performances: both the RRF and FRF of Stent A-*W*1 were bounded by those of U1 and U2, while the stiffer Stent B-*W*1 yielded values between U2 and U3. Compared to Stent B-*W*1, the gradient-stiffness Stent C-*W*1 reduced RRF and FRF by 7.0% and 5.7%, respectively. In addition, locally widening the struts to W3 improved the RRF of Stents A, B, and C by 77.2%, 52.2%, and 57.0%, with only a marginal 12% penalty in FRF.

### 3.2. Immediate Deployment Efficacy

**Figure 7:**
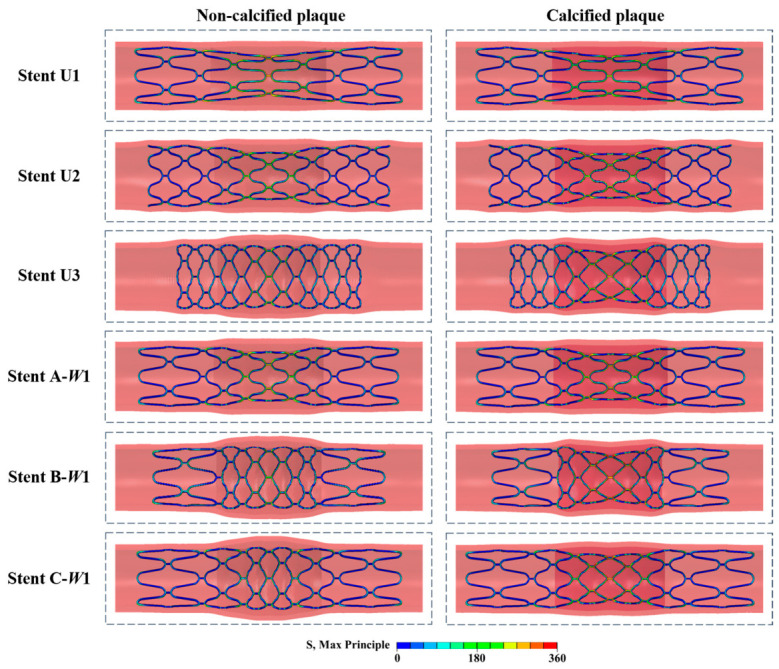
Stress distribution after stent deployment.

Figure 7 illustrates the tensile stress field after deployment, which mechanically drives the subsequent SCC. Across all designs, high tensile stresses were primarily localized at the outer curvatures of the strut crowns and links within the central stenotic segment, providing the critical radial scaffolding force against the plaque. For any identical stent, the calcified plaques induced broader and more severe high-stress zones than the non-calcified models.

The longitudinal luminal profiles reveal a mechanical trade-off in the uniform-stiffness stents. The low-stiffness Stent U1 suffered from central recoil, failing to dilate the plaque sufficiently. The higher-stiffness Stent U2 improved central support but triggered marginal over-expansion. The stiffest uniform design, Stent U3, further scaffolded the central plaque but exacerbated this severe marginal over-expansion. The optimized variable-stiffness designs resolved this trade-off: they delivered robust central radial support comparable to that of Stent U3, while suppressing marginal over-expansion to maintain a smooth luminal transition aligned with the adjacent healthy artery.

**Figure 8:**
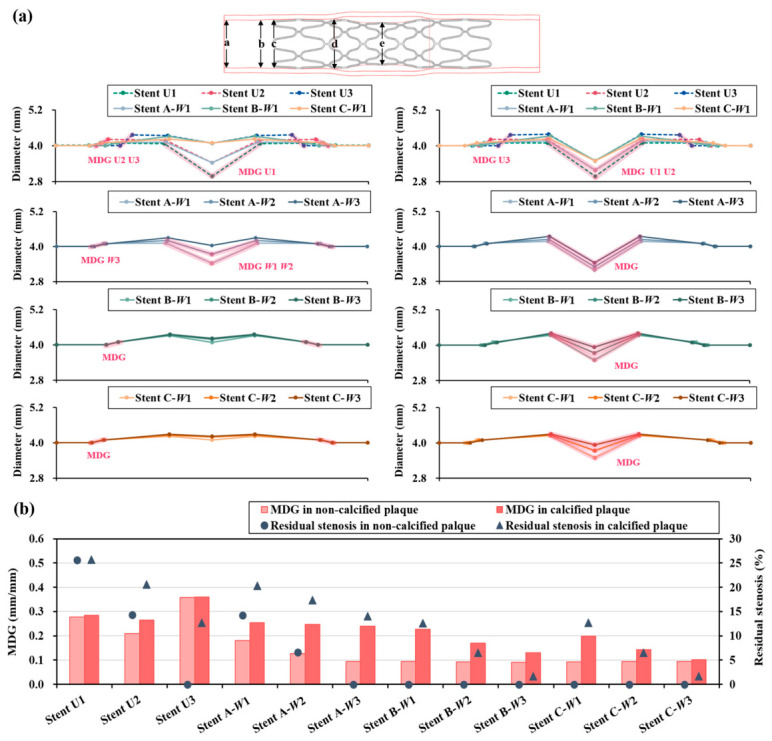
Effect of stent deployment: (**a**) Lumen diameter along the artery axis; (**b**) Lumen diameter variations and residual stenosis.

To quantify these structural advantages, five characteristic cross-sections were selected to describe the longitudinal luminal variations (Figure 8a). Assuming geometric symmetry, data extracted from the left half were mirrored to represent the entire computational domain. Compared to their uniform counterparts featuring identical central strut structures, namely Stent U2 and U3, the dual-stiffness Stents A-*W*1 and B-*W*1 eliminated marginal over-expansion while achieving equivalent central dilation at cross-section ‘e’ across both plaque models. Furthermore, while Stent U2 and U3 exhibited pronounced over-expanded along the ‘b–d’ segment, a localized outward protrusion remained evident at cross-section ‘d’ even for Stents A and B. The gradient-stiffness Stent C maintained the central luminal gain of Stent B but further mitigated this bulging at the plaque shoulder. Additionally, increasing the central strut width from *W*1 to *W*3 amplified local radial stiffness and improved central expansion, but caused the outward bulging at the plaque shoulders.

The residual stenosis rate and the maximum diameter gradient (MDG) were evaluated to quantify the expansion efficacy within the stenotic artery and the abruptness of luminal transitions, respectively (Figure 8b). Our results demonstrated that luminal expansion efficacy depended on plaque composition and local stent stiffness at the plaque position. Stent U1 failed to expand effectively, with residual stenosis exceeding 25% in both models. For the low-stiffness designs, Stents U2 and A-*W*1, residual stenosis was 14.3% and 20.6% in non-calcified and calcified plaques, respectively. Despite localized widening, Stent A-*W*3 reached 0% in the non-calcified plaque but retained 14.0% in the calcified one. Designs with higher stiffness, namely Stents U3 and B-*W*1, achieved 0% in the non-calcified model, but the residual stenosis in the calcified artery remained at 12.7%. However, with localized widening, Stent B-*W*3 reduced the residual stenosis in the calcified plaque to 1.7%. The gradient-stiffness Stent C and Stent B exhibited nearly identical residual stenosis rates across both plaque types.

The MDG was defined as the maximum change in luminal diameter between adjacent cross-sections along the axial direction. All variable-stiffness designs yielded a lower MDG (Figure 8b). Specifically, in non-calcified and calcified plaques, Stent A-*W*1 reduced the MDG by 35.2% and 11.0% compared to Stent U2, while Stent B-*W*1 reduced it by 54.9% and 14.2% compared to Stent U3. For Stents B and C in the non-calcified artery, the MDG originated solely from the geometric transition at the stent edges and was identical. In the calcified artery, the MDG occurred at the plaque core. Compared to Stent B, the gradient-stiffness Stent C further reduced the MDG across all three strut widths by 12.3%, 16.0%, and 21.7%, respectively.

### 3.3. Long-Term Service Performance

**Figure 9:**
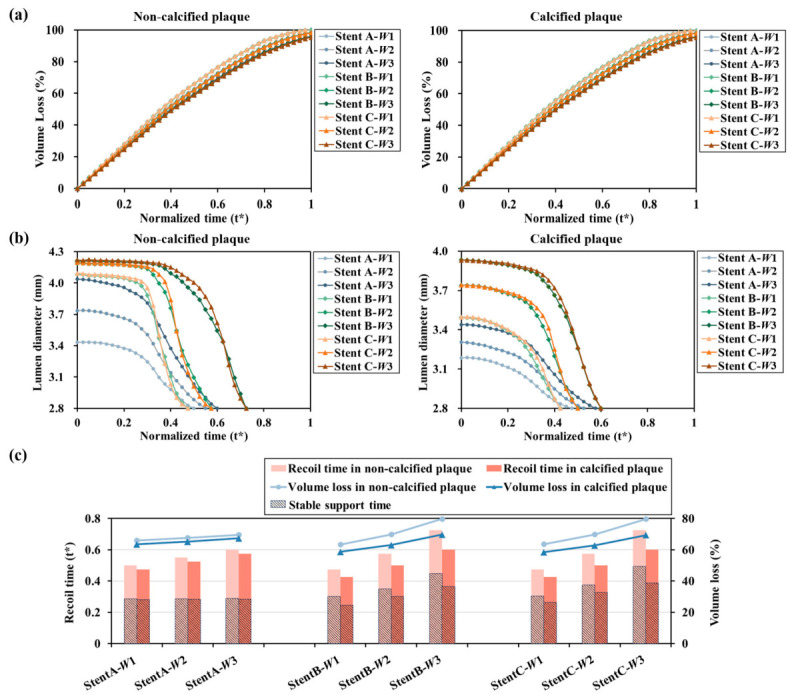
Stent degradation: (**a**) Volume of the stent; (**b**) Changes in lumen diameter; (**c**) Stent recoil time and correspond volume loss.

Degradation time t* was normalized to the time point where Stent C-*W*3 reached 90% volume loss in the non-calcified model. As shown in Figure 9a, the volume loss for all stents exhibited an initial rapid linear phase followed by a gradual deceleration. The volume loss curves for stents with the same central strut width nearly overlapped. Increasing the central strut width from *W*1 to *W*3 reduced the overall degradation rate, resulting in a downward shift of the corresponding curves.

Across all designs, the evolution of the lumen diameter exhibited a distinct two-stage profile: an initial stable support phase defined by a diameter reduction limited to 5%, followed by a gradual recoil to its pre-deployment state (Figure 9b). Despite this shared pattern, the specific degradation progress varied significantly across the different structures. Stent B-*W*1 maintained a larger lumen diameter than Stent A-*W*1 for the majority of the degradation process; however, its steeper recoil curve resulted in a marginally shorter full recoil time across both plaque models. The gradient-stiffness Stent C shared a closely matched diameter degradation profile and full recoil time with Stent B. But Stent C-*W*1 maintained a slightly higher lumen diameter at the critical inflection point where structural collapse initiated.

Quantitative analysis revealed that localized widening had a limited impact on Stent A but significantly enhances the performance of Stents B and C (Figure 9c). Furthermore, the highly rigid calcified plaques universally accelerated degradation, reducing both stable support and full recoil times across all designs. Compared to the W1 baseline, the W3 designs prolonged the full recoil times by 52.6% in non-calcified plaques and 41.2% in calcified ones. Correspondingly, the volume loss at the point of full recoil increased from 63% to 80% in the non-calcified model, and from 58% to 70% in the calcified model. Within this extended lifespan, the stable support phase also exhibited gains: as the strut width increased to W3, the stable support time for Stent B was prolonged by 48.7% and 47.7% in non-calcified and calcified plaques, respectively. While the full recoil time was identical between Stents B and C, Stent C consistently achieved a longer stable support phase. This advantage was most pronounced in the non-calcified W3 model, where C-*W*3 outlasted B-*W*3 by an additional 9.7%.

**Figure 10:**
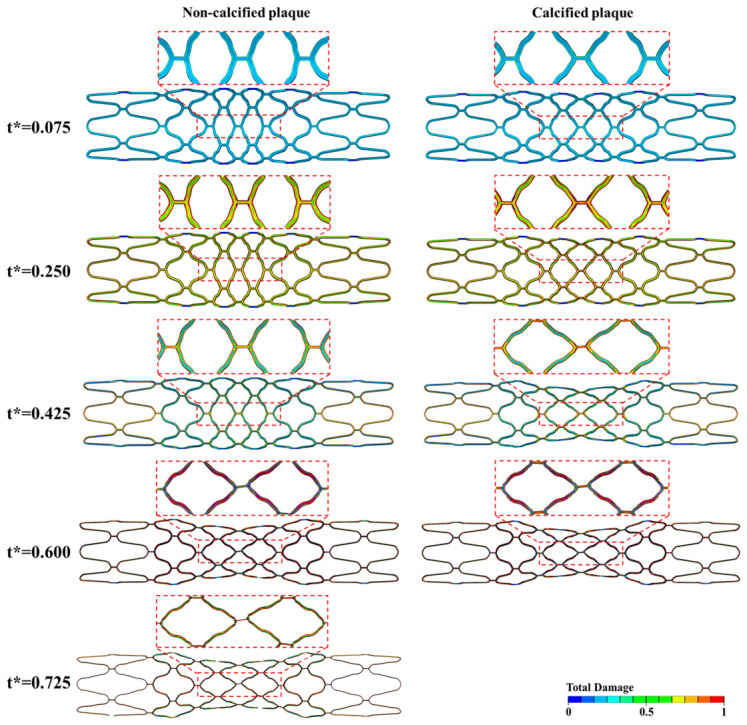
Stent degradation forms of Stent C-*W*3 in non-calcified and calcified plaques.

Given the superior service performance established above, the morphological evolution of the optimized Stent C-*W*3 was further investigated. The morphological evolution from post-deployment to the point of full recoil is shown in Figure 10. The degradation patterns remained largely consistent across both plaque characteristics. During the initial stage, elements at the strut edges experienced accelerated damage due to higher surface exposure, causing the originally square cross-sections to undergo progressive rounding and thinning. Furthermore, localized corrosion intensified at high-tensile stress zones, particularly at the junctions between the strut crowns and links. While the stent maintained global geometric continuity up until the moment of full recoil, these localized corrosion sites exhibited significantly more severe damage in the calcified plaque model. Ultimately, this stress-accelerated deterioration drove the more rapid radial recoil observed in the stiffer lesions.

## 4. Discussion

This study proposed a novel biodegradable Zn-alloy stent with a variable stiffness and evaluated its baseline mechanics, immediate deployment and long-term degradation performance via computational simulation. Our findings demonstrate that, compared to uniform stents, introducing variable stiffness achieved a more uniform lumen expansion while maintaining flexibility and radial support, and prolonged the structural scaffolding lifespan during degradation. In particular, the locally widened gradient-stiffness stent exhibited superior overall performance.

Treating focal arterial stenosis with BVSs remains a challenge, requiring the stent to provide sufficient radial support at the lesion center while maintaining structural integrity during vascular remodeling. Shortening the axial ring length to enhance radial stiffness, combined with widening the struts to further augment scaffolding and optimize stress distribution, is a conventional design strategy. However, uniformly implementing this strategy inevitably compromises overall flexibility and induces damage to the adjacent healthy arteries [32]. The variable-stiffness design selectively enhances scaffolding and widens struts exclusively at the stenotic site while utilizing compliant rings at the ends for a smooth transition, thereby effectively dilating the plaque and mitigating stress concentration without sacrificing global flexibility.

Sufficient mechanical scaffolding is essential to restore vascular patency. However, while conventional stents reliably expand non-calcified plaques, they often fail to fully dilate heavily calcified lesions. Therefore, an optimized stent design must adapt to heterogeneous plaque conditions to maintain consistent expansion capacity [33]. Clinically, the target for residual diameter stenosis is less than 10%, with an ideal goal of approaching 0% [34]. Both the widened dual-stiffness Stent B and the gradient-stiffness Stent C designs satisfied this requirement under both plaque conditions. Specifically, Stent B-*W*3 and Stent C-*W*3 achieved complete expansion in non-calcified lesions, with only approximately 1.5% residual stenosis observed in the calcified plaques (Figure 8b).

Beyond merely alleviating stenosis, normalizing local hemodynamics within the stented segment is critical. Irregular luminal geometry could potentially induce regions with reduced time-averaged wall shear stress and elevated shear rates, conditions that may raise the risk of in-stent restenosis (ISR) and stent thrombosis. While geometry does not act alone, a post-deployment luminal shape that closely mimics a healthy artery promotes favorable hemodynamics [35]. Consistent with previous studies, one of the abrupt luminal transitions occurs at the stent edges due to over-expansion [36]. While maintaining an equivalent residual stenosis at the lesion core, the variable-stiffness stents significantly attenuated this edge over-expansion compared to the uniform-stiffness stents (Figure 8a). Another abrupt luminal transition typically manifests near areas with higher residual stenosis, particularly in calcified plaques [37]. Compared with the dual-stiffness Stent B, which presented localized over-expansion at the thinner plaque segments flanking the lesion, the gradient-stiffness Stent C yielded a more uniform and smooth luminal morphology (Figure 8a).

The long-term service performance of a BVS is equally critical. The volume loss helps to quantify this degradation process. Consistent with our previous findings and those of other researchers, SCC is highly localized at the strut crowns [5]. Since this region accounts for only a small fraction of the stent volume, the vast majority of volume loss is governed by electrochemical corrosion. As the struts thinned under this uniform corrosion, the reduction in exposed surface area gradually decelerated the volume loss rate, resulting in a flatter slope in the latter half of the curves [25]. For the three variable-stiffness designs, stents with identical widths possessed nearly equivalent specific surface areas, resulting in highly overlapping volume loss curves. The localized structural widening decreases the initial specific surface area of the stent, thereby slightly slowing the volume loss (Figure 9a). Because stress corrosion severely compromises structural integrity, relying solely on volume metrics is inadequate for assessing stent degradation.

The lumen diameter serves as a direct indicator of the stent’s radial support capability during degradation. However, since vascular remodeling was not accounted for in our current model, the subsequent mechanical deterioration of the stent caused the lumen to recoil to its pre-deployment state passively. Consistent with previous in vivo imaging follow-up studies, the stents maintained stable radial support for an initial period post-deployment [38]. Comparing the structurally distinct Stents A and B with the same widths, Stent B achieved a larger initial lumen diameter. However, following the stable support period, it exhibited a steeper luminal recoil trajectory (Figure 9b). This indicates that while higher stiffness improves initial scaffolding, it correspondingly elevates local stress concentrations, which in turn accelerates stress corrosion.

Similarly, although the widened stents achieved larger initial lumen diameters, whether the resulting elevated stress and strain accelerate SCC remains a question worthy of further investigation. Experimental studies by Li et al. demonstrated that the degradation mechanisms of biodegradable zinc alloy stents are highly mechanically governed, where localized tensile stress concentrations directly dictate corrosion initiation and structural failure [39]. This finding is highly consistent with our simulation results (Figure 10). Specifically, they enhanced the stent’s radial strength by shortening the axial ring length. Their findings reveal that modifying the ring length significantly alters the spatial stress distribution; while tighter ring configurations enhance radial strength, they simultaneously exacerbate tensile stress concentrations at strut crowns, thereby increasing the propensity for SCC and premature structural failure. These observations strongly corroborate our computational rationale.

Previous studies have indicated that widening the struts of BVSs may induce excessive plastic deformation, ultimately leading to a loss of radial support or premature structural failure during degradation [40]. Although accelerated structural failure driven by these elevated stresses was not observed in our current simulations, the widened designs exhibited inconsistent improvements in radial support. For stents that failed to achieve adequate expansion initially, strut widening initially improved scaffolding. Although the support time was marginally prolonged, the recoil trajectory concurrently became slightly steeper (Figure 9b). This slight extension likely stems from the reduced initial specific surface area, and does not necessarily indicate an alleviation of SCC. Conversely, when widening was applied to a fully expanded stent, such as Stent B in non-calcified plaques, the prolongation of the support time became pronounced. This demonstrates that, provided adequate expansion is achieved, widening the struts is an effective strategy for homogenizing the stress distribution.

A larger volume loss at full recoil indicates that the struts thinned evenly, reflecting a more uniform degradation [41]. All locally widened stents showed increased volume loss at this point. Even in high-stress calcified vessels, the volume loss of the widened stents remained above 70% at full recoil (Figure 9c). This more uniform degradation better maintains stent integrity to match the vascular remodeling timeline, preventing early fracture caused by SCC.

In addition, while the gradient-stiffness Stent C exhibited a luminal recoil trajectory similar to the dual-stiffness Stent B, it achieved a further prolonged stable support time. Although this extension was relatively modest, it signifies an enhanced resistance to SCC compared to Stent B. Consequently, deploying a gradient-stiffness stent tailored to local plaque morphology yields both immediate and long-term clinical benefits.

While this study provides valuable biomechanical insights into variable-stiffness biodegradable stents, several limitations should be noted. Due to the scarcity of exact experimental SCC data for zinc alloys, the kinetic parameters were adapted from literature and extensively studied magnesium alloys. Consequently, this phenomenological model focuses on the comparative structural reliability among designs rather than predicting absolute in vivo lifespans, though the framework remains readily generalizable to other biodegradable metals.

Furthermore, complex physiological variables—including cyclic pulsatile loading, active vascular remodeling, and asymmetric plaque geometries—were simplified. This was intentional to prevent obscuring the study’s primary focus: isolating the direct coupling relationship between initial structural stiffness and SCC progression. Additionally, as static geometric metrics cannot fully characterize local hemodynamics, which closely influence the dynamic degradation process and long-term complications, subsequent fluid dynamics analyses are warranted. Therefore, while the optimized gradient-stiffness design demonstrates a prolonged scaffolding phase and superior SCC resistance, comprehensive translational assessments, such as device deliverability, material toxicity, and the risk of restenosis, fall beyond the scope of this computational framework. Further in vitro and in vivo experimental validations are essential to verify its alignment with the physiological tissue remodeling window and exact therapeutic efficacy.

## 5. Conclusions

Our findings demonstrate that the variable-stiffness stent proposed in this study enhances the radial support in the stenotic artery with minimal compromise to its overall flexibility. Furthermore, post-deployment results indicate that this design yields superior axial uniformity of the stented artery, prolongs the stable support time, and prevents premature structural failure induced by non-uniform degradation. This variable-stiffness design may provide a biomechanical foundation for the structural design and in service performance optimization of metallic BVSs.

## Figures and Tables

**Table 1:** Specific surface area of variable-stiffness stents.

Stent	Stent A			Stent B			Stent C		
	*W*1	*W*2	*W*3	*W*1	*W*2	*W*3	*W*1	*W*2	*W*3
*V* (mm^3^)	2.51	2.74	2.96	2.55	2.78	3.00	2.74	3.01	3.27
*SA* (mm^2^)	73.95	76.03	78.09	74.96	76.98	78.97	80.74	83.13	85.74
*SA*/*V* ratio (mm^−1^)	29.46	27.75	26.38	29.40	27.69	26.32	29.47	27.62	26.22

**Table 2:** Material properties of plaque [23,24].

Odgen Constants	*μ*_1_	*α*_1_	*D*_1_
Non-calcified plaque	0.093	8.17	0.43
Calcified plaque	0.32	9.25	0.13

## Data Availability

The original contributions presented in the study are included in the article; further inquiries can be directed to the corresponding author.
